# Applying of pretreatment extent of disease system in patients with hepatocellular carcinoma after curative partial hepatectomy

**DOI:** 10.18632/oncotarget.8149

**Published:** 2016-03-17

**Authors:** Guoliang Qiao, Alessandro Cucchetti, Jun Li, Matteo Cescon, Giorgio Ercolani, Guanghua Liu, Antonio Daniele Pinna, Long Li, Feng Shen, Jun Ren

**Affiliations:** ^1^ Department of Medical Oncology, Capital Medical University Cancer Center, Beijing Shijitan Hospital, Beijing, China; ^2^ Department of Hepatic Surgery, The Eastern Hepatobiliary Surgery Hospital, Second Military Medical University, Shanghai, China; ^3^ Liver and Multi-Organ Transplantation Unit, St. Orsola Hospital, Alma Mater Studiorum – University of Bologna, Bologna, Italy; ^4^ Department of Pediatric Surgery, Capital Institute of Pediatrics, Beijing, China

**Keywords:** PRETEXT system, hepatocellular carcinoma, staging systems, partial hepatectomy

## Abstract

The Pretreatment Extent of Disease System (PRETEXT) was designed for childhood liver tumors. The aim of this study was to confirm the prognostic value of the PRETEXT staging system compared with the currently and commonly used staging systems of hepatocellular carcinoma (HCC) after applying PRETEXT system in patients with HCC who underwent curative partial hepatectomy. Clinical data of consecutive patients who underwent curative partial hepatectomy were collected between February 1^st^, 2005 to December 30^th^, 2012 as the primary and internal validation cohort. Similar patients from a western hospital formed an external validation cohort. The predictive accuracy of the PRETEXT system compared with the currently used staging systems was measured by the area under the curve (AUC) on receiver operating characteristic (ROC) curve analysis. Of the 507 patients in the primary cohort, the overall median survival was 52.3 months, and the 1-year, 3-year, and 5-year overall survival rates were 83.0%, 56.8%, and 40.2%, respectively. The multivariate analysis of Cox proportional hazard regression identified INR (p=0.001), microvascular invasion (p=0.042), maximum tumor size (p=0.002) and PRETEXT staging system were independently predictors of overall survival. In the primary cohort, the AUC of the PRETEXT system was 0.702 (95% CI, 0.656 to 0.747), which was higher than the other conventional staging systems for predicting OS of HCC (P<0.01). These findings were confirmed with the internal and external validation cohorts. This study showed that the PRETEXT was a good prognostic staging system for HCC. It performed better than the conventional and commonly used staging systems in predicting survival of patients with HCC after curative partial hepatectomy.

## INTRODUCTION

Hepatocellular carcinoma(HCC) is the fifth most frequently diagnosed cancer worldwide and the second most frequent cause of cancer death [1], with the highest incidence in Asian and especially in China [2]. Partial hepatectomy remains the most commonly used curative therapy modality for HCC [3, 4]. Accurately prognostic prediction of HCC is important to facilitate screening of high risk patients and for the decision on adjuvant therapy. Many risk factors are associated with the prognosis of HCC which makes the tumor staging, prognosis estimation and choosing of therapy options complicated and difficult. Many clinical staging systems have been developed, taking into account tumor related characteristics, liver dysfunction, and general health status. These commonly used clinical staging systems included: (1) the 7th edition of TNM/AJCC classification (TNM 7th) [5]; (2) the Barcelona Clinic Liver Cancer (BCLC) staging system [6]; (3) the International Hepato-Pancreato-Biliary Association (IHPBA) staging system [7]; (4) the Okuda staging system [8]; (5) the Cancer of the Liver Italian Program (CLIP) staging system [9]; (6) the Groupe d'Etude et de Traitement du Carcinome He'patocellulaire (GETCH) staging system [10]; (7) the Chinese University Prognostic Index (CUPI) staging system [11]. Nevertheless, it remains controversial which of the established staging systems should be used as a universally applicable staging system to help improving the overall grim prognosis of HCC [12].

The Pretreatment Extent of Disease System (PRETEXT) was designed by the International Childhood Liver Tumor Strategy Group (SIOPEL) for staging and risk stratification of hepatoblastoma [13, 14]. It was based on the anatomy of the liver and depended on the assessment of the accuracy of imaging techniques preoperatively [15]. PRETEXT system was widely used as a relatively objective method to evaluate tumor extent at diagnosis. Moreover, the system had been proved to show good prognostic value for primary malignant liver tumors of childhood [16]. Many study groups also used the PRETEXT system to describe imaging findings and perform effective comparison among different staging systems of liver tumors in children. However, no researchers applied this effectively and objectively hepatic staging system to adult liver diseases.

The aim of the present study is to apply PRETEXT staging system in predicting survival of adult patients with HCC who underwent curative partial hepatectomy. The prognostic value of the PRETEXT staging system was also compared with those obtained from the currently and commonly used staging systems of HCC mentioned above.

## RESULTS

### Characteristics of the patients

The characteristics of Eastern and Western patients enrolled in two different hepatobiliary surgery units were shown in Table 1. In these three cohorts, differences among these groups were significant for most covariates. In fact, compared to the Italian, Chinese patients were younger, with predominant hepatitis B related liver disease etiology, larger tumors and better compensated liver function, while Western patients had much higher anti-hepatitis C virus (HCV) positive rate.

**Table 1:** Clinicopathological characteristics

Variable	number/value(pecent)			
	The primary cohort (n=507)	Internal validation cohort (n=233)	External validation cohort (n=293)	P values
**Age in yr(median range)**	49.6±11.2(10-77)	51.2±10.8(12-70)	63.5±9.4(40-85)	0.001
**Gender**				0.001
Male	441(87.1%)	202(86.7%)	227(77.5%)	
Female	66(12.9%)	31(13.3%)	66(22.5%)	
**HBsAg**				0.001
Positive	432(85.2%)	192(82.4%)	68(23.2%)	
Negative	75(14.8%)	41(17.6%)	225(76.8%)	
**HBeAg**				0.001
Positive	193(38.1%)	64(27.5%)	28(9.6%)	
Negative	314(61.9%)	169(72.5%)	265(90.4%)	
**Anti-HCV(+)**	0	0	202(68.9%)	0.001
**Liver cirrhosis**				0.098
Yes	367(72.4%)	176(75.5%)	232(79.2%)	
No	140(27.6%)	57(24.5%)	61(20.8%)	
**TBL(umol/l)**	15.5±8.3	15.1±7.3	16.1±8.2	0.462
**ALB (g/dl)**	39.4±6.6	39.9±6.5	37.9±4.6	0.106
**ALT(U/L)**	55.7±34.1	50.4±30.2	79.4±66.5	0.068
**INR**	1.06±0.09	1.04+0.09	1.15+1.03	0.001
PLT(*10^9^/L)	120±61	125±58	137±63	0.142
**ALP**				0.028
>130	83(16.4%)	30(12.9%)	63(21.5%)	
≤130	424(83.6%)	203(87.1%)	230(78.5%)	
**AFP(ng/ml)**				0.001
≤400	305(60.2%)	132(56.7%)	224(76.5%)	
>400	202(39.8%)	101(43.3%)	69(23.5%)	
**Blood transfusion**				0.364
Yes	128(25.3%)	70(30.0%)	82(28.0%)	
No	379(74.7%)	163(70.0%)	211(72.0%)	
**Edmondson-Steiner grade**				0.131
III or IV	315(62.1%)	160(68.7%)	198(67.6%)	
I or II	192(37.9%)	73(31.3%)	95(32.4%)	
**Tumor encapsulation**				0.341
No (no or part)	348(68.6%)	152(65.2%)	187(63.8%)	
Yes (complete)	159(32.1%)	81(34.8%)	106(36.2%)	
**Tumor diameter(<5cm)**				
**Median diameter**	5.4±3.2(1.0-12.5)	5.1±2.6(1.0-12.0)	3.9±2.1(0.7-14.0)	0.001
>5 cm	217(42.8%)	114(48.9%)	60(20.5%)	
≤5 cm	290(57.2%)	119(51.1%)	233(79.5%)	
**Microvascular invasion**				0.001
Yes	200(39.4%)	81(34.8%)	78(26.6%)	
No	307(60.6%)	152(65.2%)	215(73.4%)	
**Tumor number**				0.296
Multiple	87(17.2%)	50(21.5%)	60(20.5%)	
Solitary	420(82.8%)	183(78.5%)	233(79.5%)	
**1-year survivaL rate**	84.60%	81.40%	83.50%	
**3-year survivaL rate**	57.40%	59.40%	61.50%	
**5-year survivaL rate**	43.50%	43.90%	49.40%	

TBL: total bilirubin; ALB: albumin; ALT: alanine aminotransferase; INR: international normalize ratio; PLT: blood platelet; AKP: alkaline phosphatase; AFP: alpha-fetoprotein.

### Overall survival in the three cohorts and prognostic factors in the primary cohort

The Overall median survival times were 52.3 months(95% CI:44.4–60.2), 53.1 months(95% CI: 41.2–60.1) and 60.0 months(95% CI: 36.3–83.8) in the primary, internal and external validation cohorts, respectively. The 1-, 3-, and 5-year overall survival rates were showed in Table 1.

Univariate analysis identified that gender, liver cirrhosis, Child–Pugh classification, AFP level, the international normalized ratio(INR), tumor number, maximum tumor size, microscopic vascular invasion and PRETEXT staging system were significant prognostic factors of survival after curative resection (see Table 2). The multivariate analysis of Cox proportional hazard regression identified INR (p=0.001), microvascular invasion (p=0.042), maximum tumor size (p=0.002) and PRETEXT staging system (p=0.001) were independently predictors of overall survival.

**Table 2:** Cox proportional hazard regression analyses in the training cohort

Variable	Univariate			Multivariate		
	HR	95%CI	P value	HR	95%CI	P value
**Age in yr(median range)**	1.003	0.993-1.013	0.581			
**Male**	1.546	1.120-2.133	0.008	1.145	1.008-1.521	0.063
**HBsAg**	0.881	0.613-1.226	0.493			
**HBeAg**	0.851	0.624-1.161	0.309			
**TBL(umol/l)**	0.991	0.977-1.006	0.231			
**ALB (g/dl)**	1.159	0.784-1.771	0.459			
**ALT (U/L)**	1.001	0.996-1.006	0.812			
**INR**	0.078	0.024-0.255	0.001	0.130	0.039-0.432	**0.001**
**ALP>130U/L**	1.102	0.923-1.215	0.071			
PLT(*10^9^/L)	1.005	0.992-1.010	0.648			
**AFP>400ng/ml**	1.4	1.110-1.766	0.004	1.131	1.090-1.438	0.312
**Blood transfusion**	1.055	0.835-1.333	0.653			
**Edmondson-Steiner grade (3 or 4)**	1.049	0.840-1.310	0.671			
**Cirrhosis**	2.946	2.109-4.115	0.001	1.043	0.096-1/457	0.416
**Child–Pugh classification**	2.163	1.680-2.786	0.001	1.083	0.856-1.759	0.284
**Tumour encapsulation**	0.843	0.656-1.084	0.183			
**Tumour size(>5cm)**	2.262	1.792-2.855	<0.001	1.534	1.174-2.002	**0.002**
**Microvascular invasion**	1.414	1.106-1.807	0.006	1.294	1.010-1.659	**0.042**
**Tumor number**	1.723	1.301-2.281	<0.001	1.013	0.731-1.403	0.939
**PRETEXT system**	1.849	1.621-2.109	<0.001	1.565	1.205-2.034	**0.001**

### Staging systems in the three cohorts

**Figure 1:**
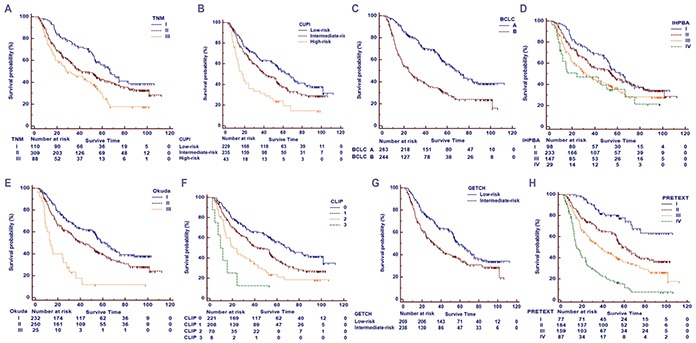
Kaplan-Meier survival curves of the primary cohort **A.** the 7th edition of TNM/AJCC classification (TNM 7th); **B.** the Chinese University Prognostic Index (CUPI) staging system; **C.** the Barcelona Clinic Liver Cancer (BCLC) staging system; **D.** the International Hepato-Pancreato-Biliary Association (IHPBA) staging system; **E.** the Okuda staging system; **F.** the Cancer of the Liver Italian Program (CLIP) staging system; **G.** the Groupe d'Etude et de Traitement du Carcinome He'patocellulaire (GETCH) staging system; H:the PRETEXT staging systems.

Among the three different cohorts, patient stratification and estimated median survival time according to the 8 staging systems were depicted in Table 3. The majority of all patients were stratified to intermediate stages of the staging systems. When looking at the individual staging system as a whole, each showed a statistically significant association with prognosis. Figure 1 showed the Kaplan-Meier survival curves stratified according to the 8 staging systems. The detail distinction between the adjacent stages of the systems was analyzed and showed in Table 3.

**Table 3:** Patient distribution and estimated median survival time according to the eight staging systems

Staging system	The primary cohort(n=507)		Internal validation cohort(n=233)		External validation cohort(n=293)	
	Median survival time(months)	P value	Median survival time(months)	P value	Median survival time(months)	P value
**AJCC TNM 7th**		<0.001		<0.001		<0.001
I	67.8	I vs. II:0.010	61.9	I vs. II:0.283	80.3	I vs. II:0.094
II	57.2	II vs. III:0.018	55.5	II vs. III:0.009	64.1	II vs. III:0.008
III	42.1		39.5		36.7	
**CUPI**		<0.001		<0.001		<0.001
Low-risk	65.2	Low-risk vs. Intermediate-risk:0.008	65.8	Low-risk vs. Intermediate-risk:0.005	77.8	Low-risk vs. Intermediate-risk:0.001
Intermediate-risk	53.2	Intermediate-risk vs. High-risk:0.001	48.9	Intermediate-risk vs. High-risk:0.547	52.3	Intermediate-risk vs. High-risk:0.171
High-risk	33.1		45.7		35.7	
**BCLC**		<0.001		0.008		0.003
A	70.4	A vs. B:<0.001	60.1	A vs. B:0.008	73.8	A vs. B:0.003
B	42.9		47		51.7	
**IHPBA**		<0.001		<0.001		<0.001
I	75.6	I vs. II:0.004	69.8	I vs. II:0.269	88.8	I vs. II:0.037
II	60.5	II vs. III:0.018	59.3	II vs. III:0.019	68.3	II vs. III:0.271
III	47.3	III vs. IV:0.091	44.9	III vs. IV:0.243	59.7	III vs. IV:0.004
IV	36.9		36.5		45.9	
**Okuda**		<0.001		<0.001		0.014
I	62.2	I vs. II:0.003	64.6	I vs. II:0.017	80.2	I vs. II:0.039
II	53.9	II vs. III:0.001	49.8	II vs. III:0.069	64.2	II vs. III:0.406
III	26		38.5		53.7	
**CLIP**		<0.001		<0.001		<0.001
0	68.5	0 vs. 1:0.001	64.4	0 vs. 1:0.030	86.7	0 vs. 1:0.032
1	52.1	1 vs. 2:0.003	52.9	1 vs. 2:0.041	68.5	1 vs. 2:0.046
2	38.3	2 vs. 3:0.073	35.9	2 vs. 3:0.273	49.2	2 vs. 3:0.735
3	15.9		27.5		49	
**GETCH**		<0.001		0.003		0.001
Low	64.3	Low vs. Intermediate: <0.001	60	Low vs. Intermediate: 0.003	76.9	Low vs. Intermediate: 0.001
Intermediate	48.8		45.2		56.4	
**PRETEXT**		<0.001		<0.001		<0.001
I	84.2	I vs. II:0.001	76.1	I vs. II:0.103	99.3	I vs. II:<0.001
II	62.3	II vs. III:0.004	63	II vs. III:0.011	65.6	II vs. III:0.006
III	51.5	III vs. IV:0.001	45.7	III vs. IV:0.006	43.4	III vs. IV:0.292
IV	28.1		28.5		32.6	

### Comparison of predictive performance of the PRETEXT system and other staging systems in the primary cohort

**Figure 2:**
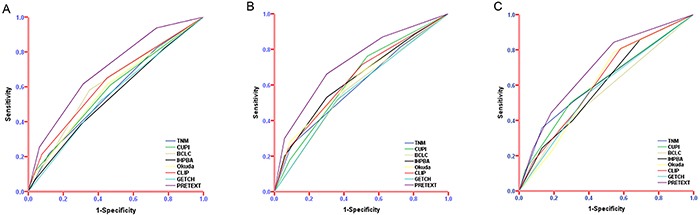
Predictive accuracy of PRETEXT system compared with other staging systems ROC analysis was displaying the ability of PRETEXT system and the currently used staging systems to predict the survival in the primary cohort **A.** internal validation cohort **B.** and external validation cohort **C.**

In the primary cohort, The performance of the PRETEXT system and the other seven conventional staging systems assessed by the likelihood ratio χ2, linear trend χ2 and the AIC test was described in Table 4. Compared with the other seven conventional staging systems, the PRETEXT system had better homogeneity (higher likelihood ratio χ2 score), discriminatory ability, and monotonicity of gradients (higher linear trend χ2 score). Also, it had a smaller AIC value, suggesting the predictive accuracy was higher. Moreover, the AUC of the PRETEXT system was 0.702 (95% CI, 0.656 to 0.747), which was higher than the other conventional staging systems for predicting survival of HCC (P<0.01, Figure 2). The AUCs of the other staging systems were 0.576 (95% CI, 0.526 to 0.626) of AJCC TNM 7th edition; 0.586 (95% CI, 0.536 to 0.636) of CUPI; 0.616 (95% CI, 0.567 to 0.665) of BCLC; 0.551 (95% CI, 0.500 to 0.601) of IHPBA; 0.593 (95% CI, 0.543 to 0.642) of Okuda; 0.624 (95% CI, 0.575 to 0.672) of CLIP and 0.553 (95% CI, 0.503 to 0.604) of GETCH.

**Table 4:** Comparison of the performance of the eight staging systems in the primary cohort

Staging system	Discriminatory ability (Linear trend x2)	Homogeneity (Likelihood ratio x2)	Akaike information criterion (AIC)
**AJCC TNM 7th**	11.342	16.967	3238.438
**CUPI**	14.85	21.48	3234.754
**BCLC**	26.77	49.422	3207.742
**IHPBA**	4.533	13.092	3242.524
**Okuda**	17.399	22.991	3232.903
**CLIP**	27.929	40.44	3217.915
**GETCH**	5.67	13.784	3241.817
**PRETEXT**	67.761	87.161	3169.021

### Validation of predictive performance of PRETEXT system

The results from the primary cohort were verified by two validation cohorts. The PRETEXT system had significantly larger AUC than those of other staging systems in the internal validation cohort(0.725 vs. AJCC TNM 7th edition: 0.599, CUPI: 0.628, BCLC: 0.582, IHPBA: 0.635, Okuda: 0.626, CLIP: 0.638 and GETCH: 0.578, P<0.05, Figure 2) and in the external cohort(0.697 vs. AJCC TNM 7th edition: 0.621, CUPI: 0.612, BCLC: 0.561, IHPBA: 0.600, Okuda: 0.609, CLIP: 0.622 and GETCH: 0.579, P<0.05, Figure 2).

## DISCUSSION

With the development of radiology and the different modalities of treatment for patients with HCC, increasing number of patients were detected HCC in relatively early stage [26], which we defined aspatients with no major vascular invasion, extrahepatic metastasis or lymph nodes spreading. Nevertheless, even in this selection of patients, the prognosis still varied since many predictors were related with the survival or the disease-free survival [27, 28]. Many staging systems had been proposed and they had been shown to have different ability to discriminate survival in HCC patients in different studies [29, 30]. Much debate still existed regarding to which prognostic staging system was the best. On the one hand, different geographic regions were attributed to various patients characteristics which led to different predictive value of the commonly used staging systems. For example, alcohol and HCV had repeatedly been identified as two leading etiologic factors for HCC in Europe studies [31], while HBV-infection was the leading etiologic factor in Chinese patients [32, 33]. On the other hand, the heterogeneity treatment options at diagnosis were associated with the lack of a consensus of different predictive value of HCC staging systems [34]. In the present study, we focused on the patients with HCC after curative partial hepatectomy, The predictive performance of the conventional staging systems were not specifically constructed for this selected group and needed to be confirmed.

After revised in 2005, PRETEXT staging system was tend to be more integral and efficient and was intended to applicable to all primary malignant liver tumor of children. It included almost all risk factors of liver tumors, such as tumor size, location, number, major vein involvement, extrahepatic abdominal disease, tumor rupture or intraperitoneal haemorrhage, distant metastases, lymph node metastases and so on [19]. More importantly, PRETEXT staging system was the only pretreatment evaluation system investigated prospectively in patients with hepatoblastoma [14]. As the widely validated effective system in predicting surgical resectability and the prognosis in liver tumor of children, we proposed to apply PRETEXT system in adult liver tumor of HCC and confirm its prognostic significance in HCC. Similarly as adult liver carcinoma TNM system used and compared with other staging systems in liver tumor of children [16, 35], we proposed to assess patients with HCC using PRETEXT system and extend it to all malignant liver tumors when its accuracy and prognostic significance were well validated in different studies. Particularly necessary to point out that since this was the first study reported PRETEXT in patients with HCC, we used the findings of perioperative exploration and the pathology report of the postoperative resection specimen rather than imaging manifestations to determine the PRETEXT staging in order to ensure the accuracy of the PRETEXT staging of patients with HCC. Meanwhile, we compared preoperative findings with the golden standard of pathologic results to further confirm the accuracy of PRETEXT staging. In the present study, we showed that 78(15.4%) of 507 tumors were incorrectly staged. This might be explained by the difficulty of distinguishing tumor and non-tumor tissues in the borderline of the different sections. The preoperative imaging findings might be used when the imaging quality and techniques improved or the error rate reduced to an acceptable range.

Our results showed that the PRETEXT system wasan independent risk factor for overall survival. Moreover, it showed superior prognostic value in both Eastern cohort and Western cohort compared with the commonly used staging systems. Multiple studies compared the staging systems in HCC and have reported different ranking of staging systems in predicting prognosis [36–39]. In our relatively selected patients, we showed that the PRETEXT staging system performed better than the conventional staging systems in the primary cohort(p<0.05), and subsequently we validated our findings in two different cohorts involving Eastern and Western patients. Among the other seven staging systems, CLIP scores showed superior performance in predicting overall survival, whereas no significance difference was observed.

There were several limitations of this study: (1) the PRETEXT system was a comprehensive and sophisticated stage for liver tumor, while we just applied part of the PRETEXT system for a selected patients with HCC. We have planned a further study to apply whole PRETEXT system to all stages of patients with HCC; (2) originally PRETEXT system was used preoperatively to evaluate prognosis of patients, thus further studies should concentrate on apply it at diagnosis; (3) since this was the first study applying PRETEXT system for patients with HCC, more studies were needed to confirm its prognostic value for HCC.

In conclusion, this study showed that the PRETEXT was a good prognostic staging system for HCC. It performed better than the conventional and commonly used staging systems in predicting survival of patients with HCC after curative partial hepatectomy.

## PATIENTS AND METHODS

### Patients

The inclusion criteria of patient selection were: (1) all patients submitted to hepatic resection as the initial treatment; (2) a preoperative ECOG criteria score of 0-1 [17]; (2) Child-Pugh class A and B; (4) histologically proven HCC in the resected specimen; (5) no evidence of extrahepatic metastasis was present at the time of surgery, and at pathologic examination did not present tumor invasion into a major branch of the portal or hepatic veins, direct invasion of adjacent organs, or spread to the lymph nodes of the hepatic hilum; (6) no tumor enucleations were included in the present study and all resections considered in the present analysis were curative resections at histology. The curative resection was defined as complete resection of tumor according to the criteria that was previously reported [18]. Applying these criteria, we prospectively collected data of consecutive patients with HCC received surgery by the same surgical team between February 1^st^, 2005 and December 30^th^, 2012 at the Eastern Hepatobiliary Surgery Hospital (EHBH) of Shanghai, China. After excluding patients with incomplete data, the final study ultimately consisted of 507 patients as the primary cohort. In the same time period 233 patients underwent resection by another surgical team in the same hospital enrolled as internal validation cohort according to the same including criteria. Moreover, another independent cohort including 293 patients were obtained from the Department of Surgery and Transplantation of the University of Bologna during February 2000 to November 2011 and using the similar inclusion criteria to serve as the external validation cohort of this study.

### PRETEXT staging system

**Figure 3:**
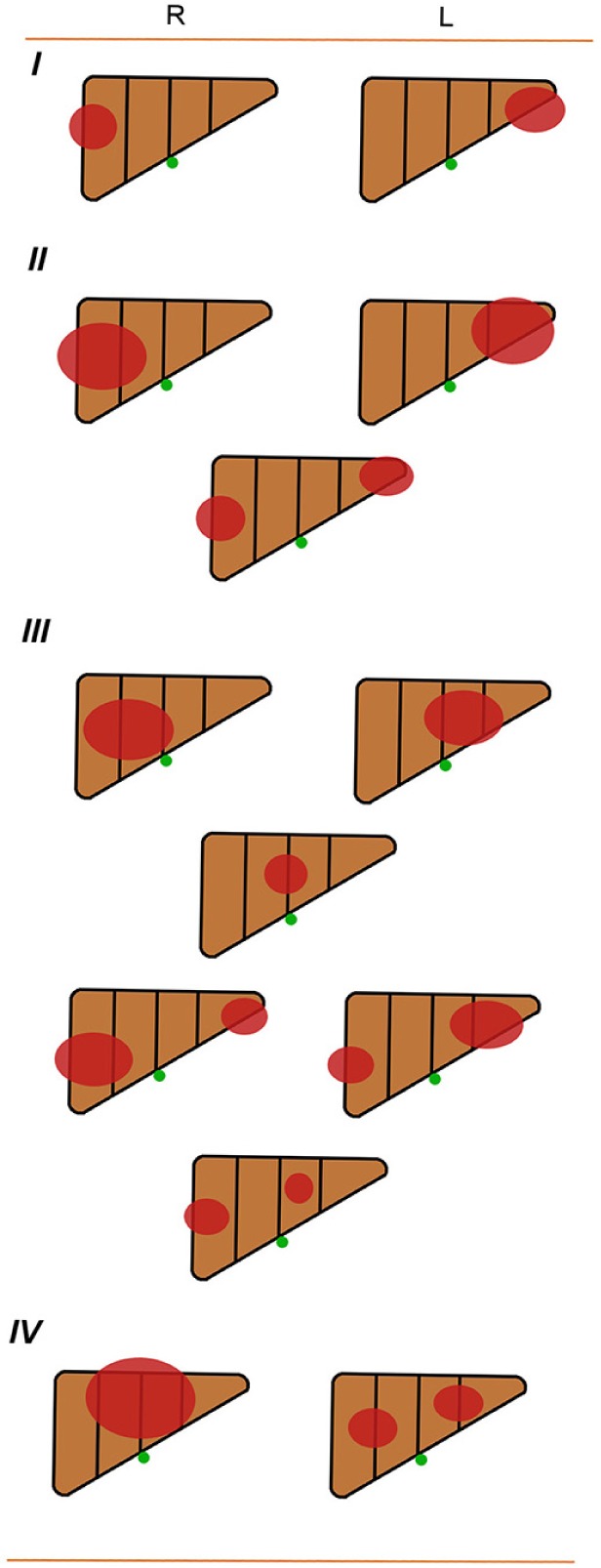
The Liver Tumor Study Group of the International Society of Pediatric Oncology(SIOP) SIOPEL-1: pretreatment extent of disease of grouping system R: right; L: left.

PRETEXT staging system, which is based exclusively on imaging at diagnosis and Couinaud's system of segmentation of the liver, divides the liver into four parts, called sections (Figure 3). The liver segments are grouped into four sections as follows: the left lobe of the liver consisting of a left lateral section (segments 2 and 3) and the medial section (segments 4); the right lobe dividing into right anterior section (segments 5 and 8) and right posterior section (segments 6 and 7). The term section is used (where other authors use segment or sector) to avoid terminological confusion. Couinaud segment 1 was not included in the original PRETEXT system. While In the 2005 revised PRETEXT system [19], tumors limited to the caudate lobe were classified as PRETEXT II. The tumor is classified into one of the following four PRETEXT categories depending on the number of liver section that are free of tumor (Figure 3). PRETEXT I (three adjacent section free of tumor); PRETEXT II (two adjacent free of tumor); PRETEXT III (one section free of tumor or two sections in one hemi-liver and one nonadjacent section in the other hemi-liver) and PRETEXT IV (no tumor free section involved). There is no change in numbering for tumors involving the caudate lobe and any other part of the liver.

The assessment of the extent of the primary tumor was performed by abdominal ultrasound and computed tomography (CT). Magnetic resonance imaging or hepatic angiography was only performed if thought necessary by the surgeon of local center. In this study, patients were staged to PRETEXT system according to the findings of perioperative exploration and the pathology report postoperatively, which could ensure the accuracy of the PRETEXT staging.

### Clinical staging systems

Collected data was used to restage all patients. This included all patients assessed by the TNM 7th edition, BCLC, IHPBA, Okuda, CLIP, GETCH and CUPI staging systems.

### Diagnosis and treatment

After a detailed history and a complete physical examination, the hepatitis B and C serology, liver function test and tumor markers examination which included alpha-fetoprotein (AFP), carbohydrate antigen 19-9 (CA19-9), and carcinoembryonic antigen (CEA) was routinely performed. Other routine investigations were chest X-ray, upper gastrointestinal endoscopy, abdominal ultrasound, contrast-enhanced computerized tomography (CT) and/or magnetic resonance imaging (MRI). A clinical diagnosis of HCC was based on the criteria of the American Association for the Study of Liver Diseases (AASLD) [20].

The type of partial hepatectomy carried out was based on the tumor size, number, location, presence/absence of cirrhosis and estimated volume of future liver remnant. As far as possible, anatomical liver resection was carried out basing on Couinaud's liver segments, sectors and hemilivers.

Histopathological study of the resected specimens was carried out independently by three pathologists who came to a consensus by discussion if there was any discrepancy.

### Follow-up

Contrast-enhanced CT scan or MRI of the abdomen was carried out once every 3 months in the first two years after surgery, and then once every 6 months thereafter. Further investigations were carried out when clinically indicated or when tumor recurrence was suspected. The diagnostic criteria for HCC recurrence were the same as used for the initial diagnosis. Overall survival (OS) was used as the primary endpoint of this study. OS was defined as the interval between partial hepatectomy and death or the last date of follow-up.

### Statistical analysis

Continuous variables were expressed as mean ± SD (standard deviation) and compared using a two-tailed unpaired Student's t test; categorical variables were compared using χ2 or Fisher analysis. Survival curves were plotted using the Kaplan–Meier method, and the log-rank test was used to determine significance [21]. Factors that were deemed of potential importance on univariate analysis were included in multivariate analyses which was performed by means of the Cox proportional-hazards model using the forward logistic regression (LR) stepwise procedure for variable selection. Homogeneity (small difference in survival among patients in the same classification within each system) was determined by likelihood ratio χ2 which was generated by the Cox proportional hazards model. The discriminatory ability of each staging system (greater difference in survival among patients in different stages within each system) was measured by linear trend χ2 [22]. Additionally, To assess potential bias in comparing prognostic systems with different numbers of stages, the Akaike information criterion (AIC) value within a Cox proportional hazard regression model was used. The AIC statistic was defined by AIC = −2 log maximum likelihood + 2*number of parameters in the model. A smaller AIC value indicated a better model for predicting outcome [23, 24]. The predictive performance of PRETEXT system and the other staging systems were measured using the area under ROC curve (AUC). AUCs were also used to compare the PRETEXT system and other staging systems using the Hanleyand McNeil method [25]. Statistical analyses were conducted with the SPSS for Windows version 18.0 release (SPSS, Inc., Chicago, IL) and ROC curve analysis were computed using MedCalcV.11.0.3.0 (MedCalc software, Mariakerke, Belgium). A value of P < 0.05 was considered significant in all the analysis.
